# The effect of lung-conduction exercise in chronic obstructive pulmonary disease: Study protocol for randomized, assessor-blind, multicenter trial

**DOI:** 10.1097/MD.0000000000019826

**Published:** 2020-05-01

**Authors:** Su Won Lee, Yee Ran Lyu, So Jung Park, Jin Young Kwak, Won Kyung Yang, Seung Hyung Kim, Weechang Kang, Ji Woong Son, In Chul Jung, Yang Chun Park

**Affiliations:** a Division of Respiratory Medicine, Department of Internal Medicine, College of Korean Medicine, Daejeon University; b Clinical Trial Center, Daejeon Korean Medicine Hospital of Daejeon University; c Institute of Traditional Medicine and Bioscience; d Department of Statistics, Hyehwa Liberal Arts College, Daejeon University; e Division of Respiratory and Critical Care Medicine, Department of Internal Medicine, Konyang University Hospital; f Department of Neuropsychology, College of Korean Medicine, Daejeon University, Daejeon, Republic of Korea.

**Keywords:** chronic obstructive pulmonary disease, Korean medicine, lung conduction exercise, pulmonary rehabilitation

## Abstract

**Background:**

Chronic obstructive pulmonary disease (COPD) is an irreversible disease characterized by cough, sputum production, and dyspnea, and has a high prevalence and mortality. Pulmonary rehabilitation (PR) is a management that improves the quality of life for COPD patients; however, PR is not readily accessible. Therefore, we developed lung-conduction exercises (LCE) that can be performed without any limitations. LCE consists of breathing, stretching, and tapping to relieve dyspnea in COPD patients.

**Methods/design:**

This randomized, assessor-blind, multicenter trial aims to recruit 54 patients with moderate and severe COPD. Subjects will be randomly allocated to a control group (only medication), an LCE group (medication + LCE, 5 times a week), or a PR group (medication + PR, 5 times a week). The 6-minute walk distance, pulmonary function tests (forced expiratory volume at 1 second, forced vital capacity, and forced expiratory volume at 1 second/forced vital capacity), modified Borg scale, modified medical research council dyspnea scale, COPD assessment test, and St. George respiratory questionnaire will be measured before starting the trial and after the 4th and 8th weeks to determine motor performance, lung function, and dyspnea.

**Conclusion:**

We aim to demonstrate that LCE is effective in improving symptoms and psychosomatic stability in COPD patients. Therefore, this trial will play an important role in fortifying the foundation of clinical application.

## 1 Introduction

Chronic obstructive pulmonary disease (COPD) is a lung disease characterized by airflow limitations that are not fully reversible.^[1]^ The global prevalence of COPD has increased from 11.7% in 2010 to 12.16% in 2015.^[2,3]^ Moreover, COPD was reported to be the fourth leading cause of death in 2000^[4]^ and it is projected to rank as the third cause by 2020.^[5]^ Thus, better practical management of COPD is needed as the socioeconomic burden of the disease increases.^[4]^

Many essential pharmacological treatments have been developed for COPD patients.^[6]^ However, medications only target the symptoms and cannot prevent the progressive decline in lung function, an essential component of the etiology of COPD.^[7]^ The drugs used for anxiety and depression, common comorbidities in COPD, are known to have had adverse effects (AEs) such as tremor, sweating, and confusion.^[8,9]^ Therefore, additional non-pharmacological treatments are required. Pulmonary rehabilitation (PR), a typical nonpharmacological treatment, is beneficial for treating depression and anxiety.^[10,11]^ However, PR also has limitations because it requires professionals and considerable time to make numerous hospital visits.^[12]^ Therefore, a self-controlling method that replaces the existing PR without these limitations is needed.

In Korean medicine, many ancient studies have demonstrated methods and exercise therapies that can treat and prevent pulmonary diseases.^[13–15]^ Especially, ⌈*Dong-Ui-Bo-Gam*⌋, an ancient medical literature approved by UNESCO as a cultural heritage in 2009, suggested *Taesikbub ((Tai Si method)* and *Lung-doyinbub(Lung guidance)*.^[14]^*Taesikbub* is a respiration method focused on taking deep breaths, and *Lung-doyinbub* is a strengthening pulmonary exercise that includes the practicing of gymnastics, tapping, and breath-holding. We developed a lung-conduction exercise (LCE) that combines *Taesikbub* and *Lung-doyinbub* and that can be used by patients in the comfort of their homes.^[16]^ This clinical trial is intended to determine the effects of LCE as a self-therapy. We planned a randomized, assessor-blind, multicenter trial to compare the effects of LCE combined with PR in COPD versus control patients.

## 2 Methods/design

### 2.1 Study design

**Figure 1:**
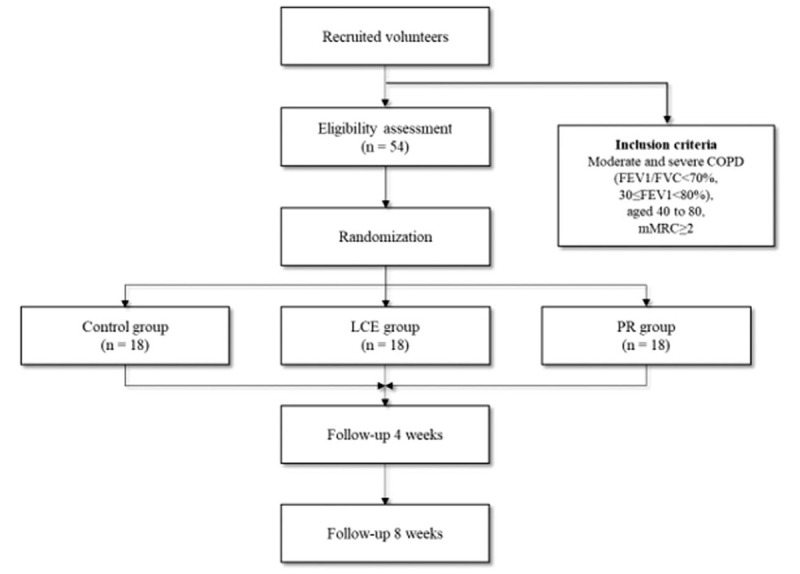
Flowchart of the study plan. Control group: medication only, LCE group: medication plus lung-conduction exercise (LCE) 5 times per wk; PR group: medication plus pulmonary rehabilitation (PR) 5 times per wk.

This randomized, assessor-blind, multicenter trial will be conducted at the Daejeon University Duncan, Korean Medicine Hospital and Konyang University Hospital in Korea. This clinical trial will consist of 8 weeks of LCE, PR, or control treatment. Assessments will be done at baseline (ie, 0-week), after 4 weeks, and 8 weeks of intervention. LCE or PR will be administered 5 times per week (Fig. 1).

### 2.2 Participants

#### 2.2.1 Inclusion/exclusion criteria

Patients with moderate and severe COPD who are diagnosed by forced expiratory volume in 1 second (FEV1)/forced vital capacity (FVC) <70% and FEV1 ≥30% but <80%, and age 40 to 80 years, are included in this trial. Participants who have complaints of difficulty in breathing at or above modified medical research council dyspnea scale (mMRC) ≥2 points and voluntarily agreed to participate in this clinical trial are included.

The exclusion criteria are as follows: patients who have serious respiratory illnesses other than COPD (eg, lung cancer, pneumonia, active tuberculosis, tuberculosis pulmonary destruction, pneumonectomy, etc); or with unstable cardiovascular disease (unstable angina, acute myocardial infarction, severe aortic stenosis, etc), and severe untreated pulmonary hypertension; or with a history of acute deterioration within 2 weeks; or have a change in FEV1 of 12% or FVC of 200 mL or more for 1 second before or after bronchodilator and asthma attack; or with other illnesses that may cause death or disability in a 1-year period (eg, cancer, heart failure, coronary artery disease, cerebrovascular disease, kidney failure, diabetes with severe complications, uncontrolled hypertension, etc); or have difficulty walking (eg, cerebrovascular disease, osteoarthritis, and serious malnutrition); or patients incapable of giving a consent or who cannot continue the test because of mental status change or other problems with intellect; or pregnant or lactating women; or alcoholics or those with a history of substance abuse; or smokers; or participants who took medication in other clinical trials within 30 days before start of this trial (based on written consent); or those with an underlying disease deemed by the investigators to be inappropriate for this trial.

#### 2.2.2 Sample size

We have divided the patients into 3 groups: control, LCE, and PR. The number of subjects is based on testing for differences in observed change after a 6-minute walk distance (6MWD) between the experimental group and the control group before the test (week 0, baseline) and after the test (week 8). In an earlier comparable clinical study, the difference was reported to be 42 with a standard deviation of 39.^[17]^ The target sample size is 18 patients per group to detect a difference of 50 m with 80% power and *a* = 0.05. To account for a probability of 20% dropout and lost data, we will recruit a total of 54 subjects.^[18]^

#### 2.2.3 Recruitment

Participants are being recruited from the Out-Patient Departments (OPDs) of the affiliated hospitals through advertising with posters and brochures. Recruitment started in November 2018.

#### 2.2.4 Participant timeline

This trial will last for 8 weeks during which LCE, PR, or appropriate controls will be administered. Participants will attend 3 assessment visits after screening and completed a series of questionnaires and other evaluations (Table 1).

**Table 1:**
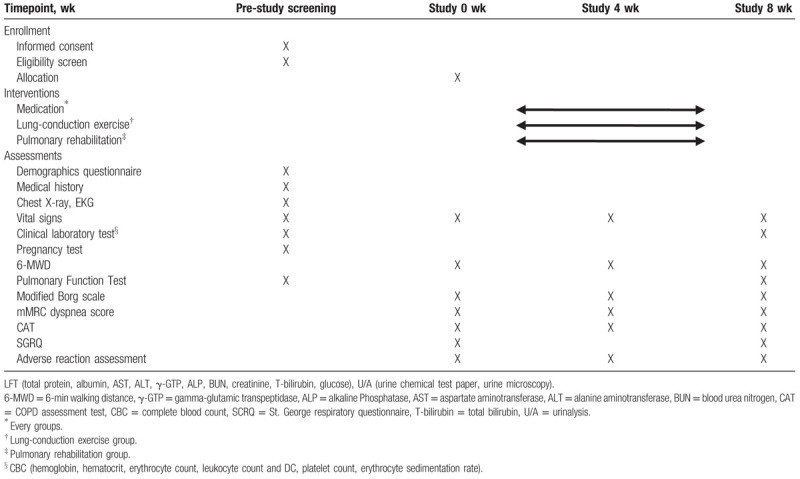
Enrollment schedule, interventions, and outcome measurements.

### 2.3 Interventions

#### 2.3.1 Control group

Patients in this group will follow only standard medications. Medications are limited to long-acting muscarinic antagonists, long-acting beta-agonists, or long-acting muscarinic antagonists and long-acting beta-agonists complexes, and short-acting beta-agonists can be used if necessary.

#### 2.3.2 PR group

Patients in this group will receive the standard PR based on the 2015 Respiratory Rehabilitation Guidelines published by the Korea Academy of Tuberculosis and Respiratory diseases.^[19]^ Patients will perform warm-up, stretching, cardiovascular exercise (using an ergometer or by treadmill walking), strength exercise, flexibility exercise, and warming down. Cardiovascular exercise is effective in increasing walking distance, strengthening cardiopulmonary function, and increasing oxygen consumption in peripheral muscles.^[20]^ Strength exercise is used for reconditioning skeletal muscles, and flexibility exercises improve chest mobilization and relaxation of postural tension.^[21]^ The intensity can be adjusted to the subject's ability. PR takes 60 minutes per day, 5 times a week for 8 weeks total (Table 2).

**Table 2:**
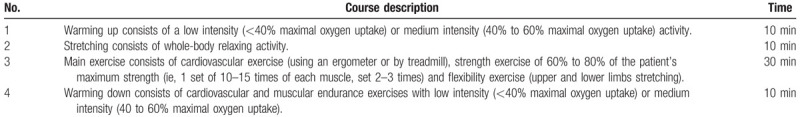
Pulmonary rehabilitation course.

#### 2.3.3 LCE group

The LCE is the Korean medicine's PR developed by the Dunsan Korean Medicine Hospital of Daejeon University after reviewing the ancient Korean Medicine literature and consulting with experts.^[16]^ In the beginning, *Taesikbub* involves taking a deep breath in and then partially breathing out, employing both diaphragmatic and pursed-lip breathing. Three times of *Taesikbub* is used to prevent airway obstruction and improve expiration because of active and prolonged efficient breathing.^[22,23]^ By closing the eyes and focusing on breathing slowly, the respiratory rate per minute is reduced and blood circulation improves, resulting in a relaxing effect.^[24]^ Subsequently, the movements of the chest and upper limbs increase the mobility of the thorax and spine and organize the movements to aid the upward and downward diaphragmatic breathing motions.^[25]^ The fist is then pounded on the left and right sides of the spine 15 times, which is similar to the percussion used for sputum discharge.^[26]^ The next steps are to activate the brain and stimulate circulation that clears the mind and promotes saliva secretion.^[27]^ Finally, after 3 more times of *Taesikbub*, the mind is stabilized, and the exercise is completed. LCE takes 20 minutes per day for 5 times a week and for a total of 8 weeks (Table 3).

**Table 3:**
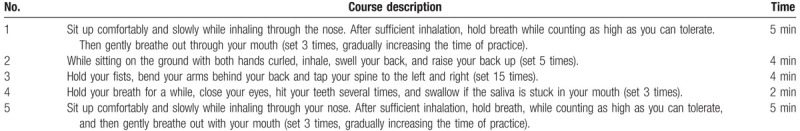
Course description of lung-conduction exercise.

### 2.4 Outcome measures

#### 2.4.1 Primary outcome measures

##### 2.4.1.1 The 6MWD

The 6MWD is one of the most widely used outcomes in PR of patients with COPD. It is also an important measure of the exercise capacity of patients with COPD because its results can provide relevant information about the patient's toleration of activities of daily living, risks for COPD exacerbations, and death.^[28]^

The primary outcome measure is the change in 6MWD after the complete trial (ie, after week 8) compared to baseline.^[29]^ The observed changes at each visit will be analyzed by repeated measures analysis of variance (ANOVA).^[17]^

The 6MWD test measures the total distance walked in 6 minutes. The test is performed using a flat, straight course of approximately 30 m. Patients will be instructed to walk as much as possible for 6 minutes while letting them know that they could rest or stop if tired. After the test, the total walking distance is calculated and recorded.^[30]^ Tests will be performed, measured, and recorded every 4 weeks (week 0, week 4, and week 8).

#### 2.4.2 Secondary outcome measures

##### 2.4.2.1 Pulmonary function test (FEV1, FVC, and FEV1/FVC)

Patients with COPD typically show a decrease in both FEV_1_ and FVC, and the presence of airflow limitation is defined by a post-bronchodilator FEV_1_/FVC <0.70.^[1]^ Therefore, we will determine whether pulmonary function was improved after the trial. Using our hospital's spirometer Vmax20, the volume of air forcibly exhaled from the point of maximal inspiration (FVC), the volume of air exhaled during the first second of this maneuver (FEV1), and the ratio of these 2 measurements (FEV1/FVC) were measured. The investigator verified the pre-test preparations with the subjects to increase the reproducibility of the test and explained that they must make every effort to ensure the correct administration. Subjects will be asked to repeat the test at least 3 times as dictated by the inspector until a suitable test with no error was done, with the reproducibility criteria met.^[31]^ Tests will be performed before and after the trials (ie, at week 0 and at week 8).

##### 2.4.2.2 Modified Borg scale

The modified Borg scale consists of 0 (no breathing difficulty) to 10 points (maximum breathing difficulty) and is a measure of respiratory distress at the time of questioning.^[32]^ The modified Borg scale can be used to evaluate various symptoms related to exercise and to assess the degree of difficulty in breathing and muscle fatigue during exercise. The scale is evaluated per 4 weeks to determine the change in scores for each visit point (week 0, week 4, week 8).

##### 2.4.2.3 mMRC score

The mMRC score, classified from 0 to 4 points of respiratory distress, is easy to use, and a highly reproducible indicator that can be effectively used to select patients for rehabilitation.^[33]^ The severity assessment is used as an assessment along with the history of COPD exacerbation and the FEV1% estimate. Tests will be performed and measured every 4 weeks (ie, at week 0, 4, and 8).

##### 2.4.2.4 COPD assessment test (CAT)

The CAT is a short and simple questionnaire for evaluating and monitoring COPD. It has fine measurement properties, with a score range of 0 to 40, suggesting a general clinical aspect and possible management considerations for the effect of COPD on scores. Therefore, it is sensitive to differences in state and should provide a valid, reliable, and standardized measure of COPD health status.^[34]^ The test is conducted every 4 weeks, and the score is recorded and the change in score at each point is measured. (ie, at week 0, 4, and 8).

##### 2.4.2.5 St. George respiratory questionnaire (SGRQ)

The SGRQ is designed to measure health-related quality of life in patients with asthma and COPD.^[35]^ Because COPD patients have poor health-related quality of life due to dyspnea, it is important to assess health-related quality of life. The questionnaire consists of 50 items and is divided into 3 areas: symptom area, activity area, and impact area. Scores range from 0 to 100, with 0 representing the best quality of life-related to health, and higher scores lower quality of life.^[36]^ The validity and reliability of the Korean version of the SGRQ has been proven, and the test will be performed before and after trial^[37]^ (week 0, week 8).

#### 2.4.3 Safety assessment

Safety assessment will be implemented by means of AEs, vital signs, and clinical laboratory tests (liver function test and routine blood and urine tests). AEs and vital signs will be recorded on a case report form at every visit, and clinical laboratory tests will be conducted before and after the clinical trial. AEs are symptoms that have not been observed before trial intervention, including unintended symptoms regardless of the trial. Investigators kept a complete record of symptoms, signs, duration, severity, relationship with the trial, measures, and outcomes of AEs. Serious adverse reactions should be reported to the principal investigator within 24 hours. The principal investigator should take action to suspend clinical trials, perform final clinical and chemical laboratory tests, and follow up until symptoms disappear.

### 2.5 Assignment of interventions

#### 2.5.1 Allocation

The independent statistician will use a random computer-generated number in the SAS Analytics Pro (SAS Institute, Cary, NC) to ensure randomization. After consent to the clinical trial, the subject identification code (random assignment number) will be assigned to those who meet the inclusion and exclusion criteria through subject suitability evaluation. Participants will be allocated to randomized and parallel groups at the same ratio of 1:1:1 for LCE or PR of the control group.

The randomization table will be maintained separately by the statistician until the trial is completed to maintain blinding. The statistician submits a written pledge not to reveal blinding. The assignment is designed according to the assignment table created by a random assignment method that can be specifically planned and reproduced in advance. The assignment table is preserved by the statistics manager, and the file protected from disclosure. All analyses will be performed using the SAS Version 9 statistical software (SAS Institute).^[38]^ Statistical significance was determined based on *a* = 0.05.

#### 2.5.2 Blinding

This is an assessor-blind trial because participants and investigators cannot be blinded during rehabilitation. The assessor should not know what type of treatment the subject is receiving and just perform the task of evaluating the validity of the subject.

### 2.6 Data management and monitoring

The investigator should be a participant in the training meeting who has the correct analysis and knowledge of the clinical study plan. Data assessment will be collected at week 0 (baseline), week 4, and week 8 (end of the trial). The investigator should keep a copy of all clinical trial-related communications, the subjects’ records, consents, and case records for at least 5 years after the end of the clinical trial in a controlled-access laboratory archive.

Clinical trial monitoring is conducted through regular site visits and telephone calls by a member of the monitoring committee to monitor the progress and regularly review and verify that it is being conducted and recorded in accordance with plans, standard work instructions, clinical trial standards, and regulations. The person in charge of monitoring will check the original subject records and data storage during the visit and consult with the investigator if any problems arise throughout the whole process of the clinical trial.

### 2.7 Statistical analysis

The target group can be divided into a full analysis set (FAS) analysis group and a per-protocol PP (PP) analysis group. The FAS analysis group is defined as the analysis group based on intention-to-treat principles. In this trial, subjects met the inclusion and exclusion criteria and were assigned randomly, and received at least 1 trial intervention when evaluating effectiveness composed of randomization groups. The PP analysis group consisted of subjects who had completed the entire course of the trial without violating the protocol. According to The International Council for Harmonisation of Technical Requirements for Pharmaceuticals for Human Use (ICH) E9 (Statistical principles for clinical trials), FAS is the analysis target group closest to the concept of the “assigned analysis” principle, which allows for the exclusion of the minimum number of subjects from the analysis for justifiable reasons from all randomized subjects.^[38]^

Therefore, effectiveness evaluation is mainly based on FAS analysis based on the intention-to-treat principle, and PP analysis is the secondary analysis. The primary efficacy outcome measure, the 6MWD, is analyzed by repeated measures ANOVA to control groups with score changes before and after the trial and observed at each visit. The analysis of secondary efficacy outcome measures were as follows: pulmonary function test (FEV1, FVC, FEV1/FVC) and SGRQ by ANOVA including its baseline value; modified Borg scale, mMRC score, and CAT by linear mixed models.

Safety evaluation was conducted in a group of subjects who received 1 or more interventions, and the assessor confirmed at least 1 safety-related data by visit or call after the trial intervention. A comparison of the number of AEs associated with the trial is performed using the Kruskal–Wallis test, and group comparisons of the proportion of subjects who experienced 1 or more AEs are analyzed using the Pearson *χ*^2^ or Fisher exact test.

Statistical significance including primary outcome measures, secondary outcome measures, and safety evaluations is set at 5% significance level.^[38]^

## 3 Ethics and dissemination

This clinical trial protocol is in compliance with all applicable regulations, including the ICH Good clinical practice (GCP) Guidelines, the Helsinki Declaration (Seoul 2008), the Korean GCP Guidelines, the Korean Pharmaceutical Affairs Law, the Institutional Review Board (IRB), and data protection regulations.^[39]^

The clinical trial protocol was approved by the IRB of Daejeon University Dunsan Korean Medicine Hospital and Konyang University Hospital (Approval number; DJDSKH-18-BM-19, KYUH-2018-10-014-015) including protocol, written patient consent, consent form, patient registration procedure (eg, advertising), written information provided to the patient, and pledge to comply with GCP requirements before commencement of the trial. When revising the protocol, the date of revision, the reason for the revision, and the details of the revision shall be recorded, reported to the IRB, and then preserved. The investigators should not conduct clinical trials contrary to the protocol, except where immediate risks to the subject need to be eliminated.

Patient Informed Consent will be written before the subject decides to participate in the study. The investigators provided all the information relevant to the clinical trial, including the benefits and risks of participating in this study, and the subjects signed a document containing all the instructions for the subjects. The investigators should verify that the subjects voluntarily participated in the clinical trial. A copy of the originally signed and dated consent form will be kept and another copy will be sent to the patient's legal representative.

Records on the subject's identity will be kept confidential even when the results of the study are published. All documents related to clinical trials, such as case records, should be recorded and distinguished by subject identification code, not name. However, monitors and inspectors involved in this clinical trial may view the subject's records for the purpose of monitoring and managing the progress of the trial. In addition, the document may be reviewed or copied in order to verify the subject's charts and case record records in the national legislation. All documents will be kept confidential in a controlled-access laboratory archive.

## 4 Discussion

COPD is a disease with a high prevalence and mortality worldwide.^[2]^ This trend is expected to continue because of the increasing aging population and the preponderance of risk factors. COPD is predicted to rank as 4th cause of death in 2030.^[40]^ Since COPD involves irreversible lung parenchyma destruction, it is important to alleviate symptoms and maximize the remaining pulmonary function. As shown, PR is suitable for daily management^[41]^ and it has recently seen much attention because it can be used to improve emotional changes such as depression, muscle weakness, and weight loss due to poor exercise ability and relative social isolation, events not properly treated with currently available medications.^[41]^ However, the current status of PR in Korea is limited by cost, transportation inconvenience, labor, and facilities.^[42]^ Therefore, a PR method based on Korean medicine, that is, the LCE was developed.^[16]^ Because the LCE consists of simple movements and is available regardless, it is suitable for daily self-treatment, especially of older patients. We anticipate that LCE will be effective, via respiratory meditation, in emotionally stabilizing patients as well as symptomatically relieving symptoms by increasing diaphragmatic elevation and force, increasing ventilation efficiency, thoracic movements, and sputum discharge.^[22–27]^

This is the first clinical trial for demonstrating the effect of Korean medicine's PR. In addition, we examine the effects when compared with existing standard PR. Despite the expected findings of this study, there is a limitation in that the amount of exercise is not sufficient for a single rehabilitation exercise, and the results may not be entirely due to LCE. Therefore, movements that are specific to the individual patient can be added and modified. Overall, this study will help determine the clinical effect and safety of LCE and secure the utility of Korean medicine PR exercise.

## Author contributions

These authors contributed equally to this work: Su Won Lee, Yee Ran Lyu.

## References

[1] Rabe KF Hurd S Anzueto A Global strategy for the diagnosis, management, and prevention of chronic obstructive pulmonary disease: GOLD executive summary. Am J Respir Crit Care Med 2007;176:532–55. 17507545 10.1164/rccm.200703-456SO

[2] Varmaghani M Dehghani M Heidari E Global prevalence of chronic obstructive pulmonary disease: systematic review and meta-analysis. East Mediterr Health J 2019;25:47–57. 30919925 10.26719/emhj.18.014

[3] Adeloye D Chua S Lee C Global and regional estimates of COPD prevalence: Systematic review and meta–analysis. J Glob Health 2015;5:020415. 26755942 10.7189/jogh.05-020415 PMC4693508

[4] Lopez A Shibuya K Rao C Chronic obstructive pulmonary disease: current burden and future projections. Eur Respir J 2006;27:397–412. 16452599 10.1183/09031936.06.00025805

[5] Murray CJ Lopez AD Alternative projections of mortality and disability by cause 1990-2020: Global Burden of Disease Study. Lancet 1997;349:1498–504. 9167458 10.1016/S0140-6736(96)07492-2

[6] Gómez FP Rodriguez-Roisin R Global initiative for chronic obstructive lung disease (GOLD) guidelines for chronic obstructive pulmonary disease. Curr Opin Pulm Med 2002;8:81–6. 11845001 10.1097/00063198-200203000-00001

[7] Montuschi P Pharmacological treatment of chronic obstructive pulmonary disease. Int J Chron Obstruct Pulmon Dis 2006;1:409–23. 18044097 10.2147/copd.2006.1.4.409 PMC2707800

[8] Rossi S Australian Medicines Handbook. Australian Medicines Handbook Pty Ltd: Sydney; 2006.

[9] Eiser N Harte R Karvounis S Effect of treating depression on quality-of-life and exercise tolerance in severe COPD. COPD 2005;2:233–41. 17136950

[10] McCarthy B Casey D Devane D Pulmonary rehabilitation for chronic obstructive pulmonary disease. Cochrane Database Syst Rev 2015;(2):CD003793. 25705944 10.1002/14651858.CD003793.pub3 PMC10008021

[11] Paz-Díaz H De Oca MM López JM Pulmonary rehabilitation improves depression, anxiety, dyspnea and health status in patients with COPD. Am J Phys Med Rehabil 2007;86:30–6. 17304686 10.1097/phm.0b013e31802b8eca

[12] Keating A Lee A Holland AE What prevents people with chronic obstructive pulmonary disease from attending pulmonary rehabilitation? A systematic review. Chron Respir Dis 2011;8:89–99. 21596892 10.1177/1479972310393756

[13] Hong W Kyojung Huangje Naegyeong Yeongchu. 1985;Seoul: Dongyang Medicine Institute, 198.

[14] Heo J. Dong-Ui-Bo-Gam. Hadong: Donguibogam Publish. 2005.

[15] People's Medical Publishing House (PMPH), So WB Jebyeong Wonhuron. 1988;Beijing, 143–155.

[16] Yee Ran Lyu JJP So Jung P Eun Jung L Application of Taesikbub and Lung-doyinbub in ⌈Dong-Ui-Bo-Gam⌋ as a Korean traditional pulmonary rehabilitation exercise. J Korean Med 2018;39:41–50.

[17] Kaminsky DA Guntupalli KK Lippmann J Effect of yoga breathing (pranayama) on exercise tolerance in patients with chronic obstructive pulmonary disease: a randomized, controlled trial. J Altern Complement Med 2017;23:696–704. 28714735 10.1089/acm.2017.0102 PMC5610410

[18] Chow S-C Shao J Wang H Sample Size Calculations in Clinical Research. London: Chapman and Hall/CRC; 2017.

[19] Charususin N Gosselink R Decramer M Inspiratory muscle training protocol for patients with chronic obstructive pulmonary disease (IMTCO study): a multicentre randomised controlled trial. BMJ Open 2013;3:e003101. 10.1136/bmjopen-2013-003101 PMC3740252 23921069

[20] Bianchi L Roca J Paathophysiology of Exercise and Exercise Assessment. Pulmonary Rehabilitation. London: Hodder Arnold; 2005.

[21] Neumann DA Kinesiology of the Musculoskeletal System; Foundation for Rehabilitation. Missouri: Mosby & Elsevier; 2010.

[22] Van der Schans C De Jong W Kort E Mouth pressures during pursed lip breathing. Physiother Theory Pract 1995;11:29–34.

[23] Gosselink R Breathing techniques in patients with chronic obstructive pulmonary disease (COPD). Chronic Respir Dis 2004;1:163–72. 10.1191/1479972304cd020rs 16281658

[24] Larkey L Jahnke R Etnier J Meditative movement as a category of exercise: implications for research. J Phys Act Health 2009;6:230–8. 19420401 10.1123/jpah.6.2.230

[25] Leelarungrayub D Chest mobilization techniques for improving ventilation and gas exchange in chronic lung disease. Chronic Obstruct Pulm Dis-Curr Concepts Pract 2012 400–22.

[26] Yohannes AM Connolly MJ A national survey: percussion, vibration, shaking and active cycle breathing techniques used in patients with acute exacerbations of chronic obstructive pulmonary disease. Physiotherapy 2007;93:110–3.

[27] Cho S-Y Shin A-S Na B-J Brain activity associated with memory and cognitive function during jaw-tapping movement in healthy subjects using functional magnetic resonance imaging. Chin J Integr Med 2013;19:409–17. 23263997 10.1007/s11655-012-1187-7

[28] Puhan MA Mador M Held U Interpretation of treatment changes in 6-minute walk distance in patients with COPD. Eur Respir J 2008;32:637–43. 18550610 10.1183/09031936.00140507

[29] Holland AE Hill CJ Jones AY Breathing exercises for chronic obstructive pulmonary disease. Cochrane Database Syst Rev 2012;10:CD008250. 23076942 10.1002/14651858.CD008250.pub2 PMC11371308

[30] ATS Committee on Proficiency Standards for Clinical Pulmonary Function Laboratories. ATS statement: guidelines for the six-minute walk test. Am J Respir Crit Care Med 2002;166:111–7. 12091180 10.1164/ajrccm.166.1.at1102

[31] Enright PL Lebowitz MD Cockroft DW Physiologic measures: pulmonary function tests. Am J Respir Crit Care Med 2012;149:S9–18. 10.1164/ajrccm/149.2_Pt_2.S9 8298772

[32] Borg GA Psychophysical bases of perceived exertion. Med Sci Sports Exerc 1982;14:377–81. 7154893

[33] Fletcher CM Elmes PC Fairbairn AS Significance of respiratory symptoms and the diagnosis of chronic bronchitis in a working population. Br Med J 1959;2:257–66. 13823475 10.1136/bmj.2.5147.257 PMC1990153

[34] Jones P Harding G Berry P Development and first validation of the COPD assessment test. Eur Respir J 2009;34:648–54. 19720809 10.1183/09031936.00102509

[35] Jones P Quirk F Baveystock C The St George's respiratory questionnaire. Respir med 1991;85:25–31. 1759018 10.1016/s0954-6111(06)80166-6

[36] Ferrer M Villasante C Alonso J Interpretation of quality of life scores from the St George's Respiratory Questionnaire. Eur Respir J 2002;19:405–13. 11936515 10.1183/09031936.02.00213202

[37] Kim YS Byun MK Jung WY Validation of the Korean version of the St. George's respiratory questionnaire for patients with chronic respiratory disease. Tuberculosis and Respira Dis 2006;61:121–8.

[38] Lewis JA Statistical principles for clinical trials (ICH E9): an introductory note on an international guideline. Stat Med 1999;18:1903–42. 10440877 10.1002/(sici)1097-0258(19990815)18:15<1903::aid-sim188>3.0.co;2-f

[39] Guideline IHT Guideline for good clinical practice. J Postgrad Med 2001;47:199–203. 11832625

[40] Mathers CD Loncar D Projections of global mortality and burden of disease from 2002 to 2030. PLoS Med 2006;3:e442. 17132052 10.1371/journal.pmed.0030442 PMC1664601

[41] Nici L Donner C Wouters E American thoracic society/European respiratory society statement on pulmonary rehabilitation. Am J Respir Crit Care Med 2006;173:1390–413. 16760357 10.1164/rccm.200508-1211ST

[42] Diseases TKAoTaR. Consensus Document on Pulmonary Rehabilitation in Korea 2015. Seoul: The Korea Academy of Tuberculosis and Respiratory Diseases; 2015.

